# A systematic review and meta-analysis of digital interventions targeting lifestyle factors in patients with hypertension

**DOI:** 10.1038/s41371-025-01051-3

**Published:** 2025-08-05

**Authors:** Alexandra Lindsay-Perez, Rebecca Jurdon, Thomas King, Lydia Koffman, Nia Roberts, Richard J. McManus, David McCartney

**Affiliations:** 1 Ageing and Movement Research Group, Population Health Sciences, Bristol Medical School, University of Bristol, Bristol, UK; 2 Frimley Park Hospital, Frimley Health NHS Foundation Trust, Surrey, UK; 3 Northwick Park Hospital, London North West University Healthcare NHS Trust, Harrow, UK; 4 John Radcliffe Hospital, Oxford University Hospitals NHS Foundation Trust, Oxford, UK; 5 Bodleian Health Care Libraries, University of Oxford, Oxford, UK; 6 Brighton and Sussex Medical School, University of Brighton and University of Sussex, Brighton, UK; 7 School of Medicine and Biomedical Sciences, University of Oxford, Oxford, UK

**Keywords:** Renovascular hypertension, Lifestyle modification, Clinical trials

## Abstract

Hypertension is a major risk factor for cardiovascular disease, for which the management involves both lifestyle modification (diet, exercise etc) and medication. Digital interventions (mobile applications, websites, and SMS messages) are being developed to facilitate lifestyle change, but their effectiveness remains uncertain. This review aimed to establish whether digital interventions targeting lifestyle factors are effective in reducing blood pressure in individuals with hypertension. A systematic search was run through MEDLINE, EMBASE and the Cochrane Library. 5302 records were screened for eligibility and data on the primary outcome (systolic blood pressure (SBP)) and secondary outcomes (diastolic blood pressure (DBP) and change in lifestyle factors) were extracted from eligible papers. Where sufficient data were available, meta-analysis was undertaken using a random effects model. 17 randomised controlled trials were eligible for inclusion (3040 patients). 12 studies were suitable for meta-analysis. Lifestyle change mediated by digital interventions were associated with a larger SBP reduction than controls (mean difference (MD) −2.91 mmHg; 95% confidence interval (CI) −4.11, −1.71; p value (p) <0.0001). A significant difference was also seen in DBP reduction between groups (MD −1.13 mmHg; CI −1.91, −0.35; *p* = 0.005). Reporting of other secondary outcomes relating to lifestyle change was too heterogenous for meta-analysis. Digital interventions targeting lifestyle factors were associated with an improvement in blood pressure in patients with hypertension, but interpretation of the results is limited by significant heterogeneity between studies. Further research is required to understand which lifestyle factors, when targeted with digital interventions, result in maximal blood pressure reduction.

## Introduction

Hypertension is a key risk factor for the development of cardiovascular disease and therefore is a major contributor to global disease burden [1, 2]. It is estimated that a third of adults in England have hypertension (most commonly defined as a clinic blood pressure (BP) reading >140/90 mmHg, or home or ambulatory blood pressure reading >135/95 mmHg [3]). Reducing blood pressure is correlated with reduced mortality [4] – a 10 mmHg reduction in BP can lead to a 41% reduction in stroke and a 22% reduction in coronary heart disease [5].

Blood pressure lowering can be achieved through lifestyle modification [6, 7]. This includes reduced salt intake, increased physical activity, weight loss, smoking cessation, and reduced alcohol consumption. Multi-component lifestyle interventions have demonstrated effective blood pressure reduction in hypertension [8]. As a result, both NICE and European hypertension management guidelines recommend lifestyle modification prior to or alongside anti-hypertensive medication [9, 10]. Despite evidence for the efficacy of lifestyle interventions in BP management, there is often little improvement in lifestyle factors following a diagnosis of raised blood pressure [11].

Digital interventions have been used to facilitate behaviour change, and thereby improve management, in a range of conditions [12]. Evidence from randomised controlled trials and systematic reviews supports the use of digital interventions to modify risk factors for cardiovascular disease [13], and specifically for hypertension [14]. In hypertension, digital interventions have the potential to indirectly improve blood pressure by enhancing lifestyle modification. They may also be more cost-effective than non-digital equivalents, as they can be delivered at large scale at low cost. However, the digital interventions used in hypertension trials are heterogenous and often target multiple aspects of disease management including medication adherence, lifestyle change and remote monitoring.

This systematic review aimed to establish whether digital interventions targeting lifestyle behaviour modification are effective for the reduction of blood pressure in individuals with hypertension.

## Methods

The protocol for this systematic review and meta-analysis was prospectively registered with the Prospero Database of Systematic Reviews (CRD42021292206).

### Search strategy

The search strategy was developed in collaboration with a healthcare librarian with expertise in systematic review search (see Figure, Supplementary Digital Content (SDC) 1, outlining search strategy). The following databases were searched for relevant randomised controlled trials up to 24 December 2021: MEDLINE(OvidSP) (1946 to 24 December 2021), Embase(OvidSP) (1974 to 24 December 2021) and the Cochrane Central Register of Controlled Trials (CENTRAL) via Cochrane Library, Wiley [Issues 12 of 12, December 2021].

Animal studies, conference abstracts, and publications not in the English language were excluded from the search. Publications before January 2000 were excluded as digital technology prior to that date would no longer be relevant. A search string developed by EPOC (https://zenodo.org/records/5106292) was applied to filter by study design. The authors searched references of included full-text papers to identify other relevant studies. Study protocols recovered by the search which met the inclusion criteria were searched on PubMed to identify any reported results. If none were found, the protocols were excluded.

### Inclusion criteria

Duplicate studies were removed from the search result. Titles and abstracts of remaining studies were screened independently by two review authors against the following inclusion criteria:

- Population – Adults (>18 years of age) with a diagnosis of hypertension (blood pressure ≥140/90 mmHg), without other cardiovascular disease, diabetes mellitus, chronic kidney disease, or a secondary cause of hypertension.
- Intervention – Digital interventions targeting lifestyle factors (exercise, diet, weight loss, smoking) to lower blood pressure, where the intervention was used for a minimum of 3 months. Digital interventions were defined as phone-based interventions (text messages, mobile applications, online messaging groups), computer-based interventions (emails, websites, software) or wearable digital monitors (where the results of the monitoring were used to guide lifestyle modification).
- Comparator – Usual care, placebo, or alternative intervention differing in nature or intensity.
- Outcomes – The primary outcome was change in systolic blood pressure (mmHg) from baseline to last available follow-up. Secondary outcomes were change in diastolic blood pressure, BMI, physical activity, diet and smoking from baseline to last available follow-up.

Studies with interventions primarily targeting medication adherence or involving titration of anti-hypertensive medications were excluded. Where studies had multiple intervention groups, with only one intervention group meeting the inclusion criteria, (e.g. other intervention groups medication-focused intervention), data from groups relevant to the research question was included. We excluded studies where the digital intervention was used only to facilitate a live interaction between a patient and healthcare professional, as we were specifically interested in whether digital interventions that are likely to be low-cost and scalable still mediate a blood-pressure lowering effect. Studies where the digital intervention only involved blood pressure self-monitoring, with no lifestyle advice, were also excluded. Only randomised controlled trials were included.

The full text of articles not excluded through title and abstract screening were independently assessed for inclusion by two review team members. Where disagreements between two reviewers occurred over inclusion of a paper a third review author was involved to reach a consensus.

### Data extraction

Data were independently extracted by two review authors. A standardised form was used to extract data from included studies. Background details, intervention details and data for bias assessment were extracted. Data were extracted for the primary outcome and for the secondary outcomes where available. Where discrepancies occurred in data extraction between two review authors, a third review author checked to confirm the correct figures. Where there was significant missing data, some authors were contacted to try and access this data.

### Quality assessment

The quality and risk of bias of included studies were independently assessed by two review authors using the Cochrane Risk of Bias 1 tool [15]. The Risk of Bias 1 tool was used as the review protocol was determined prior to the implementation of the Risk of Bias 2 tool and its integration into Revman [16]. Domains considered included random sequence generation, allocation concealment, blinding of participants and personnel, blinding of outcome assessment, incomplete outcome data, and selective reporting. For each domain, studies were ranked as either low risk of bias, high risk of bias, or unclear risk. Overall bias was considered on the basis of the lowest scoring domain.

### Data synthesis

A meta-analysis was undertaken to compare the mean difference in systolic and diastolic blood pressure change from baseline to end point between intervention and control groups. Meta-analysis was performed using the inverse variance statistical model in Review Manager (RevMan) software version 5.4. Given the clinical heterogeneity between studies, a random-effects model was used. Studies reporting a mean change in blood pressure for intervention and control group were included in the meta-analysis. Studies where mean change in BP could be calculated from other data reported in the study (such as baseline and end point values, p values) using the methods in Cochrane Handbook for Systematic Review of Interventions [17] were also included in the meta-analysis. The I^2^ and τ^2^ statistic was used to measure heterogeneity amongst studies. Pre-specified sub-group analyses were performed determined by intervention type, sample size, study duration, and bias risk.

Of the secondary outcomes, only change in diastolic blood pressure was reported with sufficient consistency to allow meta-analysis of mean difference from baseline to end point between groups. Results for BMI, weight, alcohol intake, salt intake, tobacco smoking and fruit/vegetable intake were therefore reported narratively.

## Results

### Search results

**Fig. 1:**
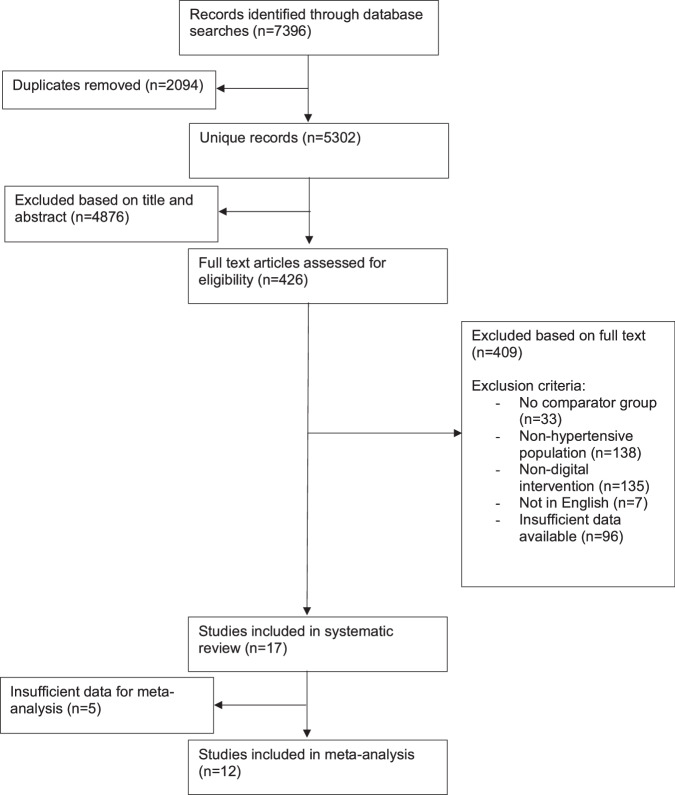
Flowchart of included studies.

Following de-duplication, the database search resulted in 5302 titles and abstracts (Fig. 1). After review based on titles and abstracts, 426 full text articles were assessed for eligibility, leaving 17 studies included in the final analysis. 12 of these had sufficient data for meta-analysis.

### Study characteristics

Characteristics of the 17 included studies are outlined in Table 1. The studies were conducted in a range of countries. Most studies were conducted in outpatient [18–25] or community [26–31] clinics.

**Table 1:** Comparison of included study characteristics.

Study	Country	Setting	Age (years)	Population	n		Target behaviour	Intervention	Control	Intervention duration (months)
					Intervention	Control				
Green [24]	US	Outpatient clinic	25–75	Mean BP > 140/90 mmHg after two screening visits	246	247	Exercise	Home BPM and website to log results. Resources for lifestyle change on website.	Pamphlet given about hypertension and advised to speak to their doctor	12
Bennett [21]	US	Outpatient clinic	25–65	Diagnosis of HTN, on anti-hypertensive medication	51	50	Diet, exercise	Regular sessions with health coach for lifestyle change, access to online self-monitoring tool	Printed weight loss materials and standard care	3
Golshahi [23]	Iran	Outpatient clinic	>18	Diagnosis of hypertension (BP > 140/90 mmHg) or screening visit BP > 140/90 mmHg	45	45	Diet, exercise, smoking cessation, medication adherence	Regular SMS messages with lifestyle advice	Standard care	8
Kim [34]	US	Not specified	>18	Recent insurance claim relating to hypertension	52	43	Not specified	Home BPM and access to online platform. Nurses able to give recommendations via platform	Access to disease management online program without nursing advice	6
Liu [25]	Canada	Outpatient clinic	35–74	Diagnosis of hypertension (BP > 140/90 mmHg)	37	39	Diet, exercise	Regular emails with access to lifestyle change resources	Weekly email newsletter without resources	4
Skolarus [27]	US	Community clinic	>18	African-Americans with BP > 140/90mmhg at screening	41	32	Diet, exercise	Home BPM. Regular SMS messages with lifestyle advice and reminders to monitor.	Written American Heart Association materials	6
Li [26]	China	Community clinic	45–70	Diagnosis of hypertension	110	143	Diet, exercise, smoking cessation, alcohol intake	Once weekly health education via ‘WeChat’ social media. Feedback on self-reported BP and groups available for discussion	Standard care	6
Meurer [32]	US	Emergency Department	Not specified	BP > 140/90 mmHg at screening visit	14	16	Diet, exercise, medication adherence	Home BPM provided. Regular SMS messages with reminders to monitor BP and health behaviour advice	Received BP cuff and HTN brochure. Instructed to follow up with own doctor	3
Rehman [19]	Pakistan	Outpatient clinic	25–65	SBP 140–160 mmHg, DBP 90–100 mmHg over the last 2 years	Not specified	Not specified	Diet, exercise	Educational talk in clinic. Home BPM. Regular SMS messages with reminders to monitor and lifestyle advice	Educational talk in clinic, then standard care	3
Borgstrom [28]	Sweden	Community clinic	40–80	Diagnosis of hypertension	29	28	Diet, exercise, smoking cessation	Regular text messages with lifestyle advice	Standard care	6
Jahan [29]	Bangladesh	Community clinic	35–71	Diagnosis of hypertension	204	208	Diet, exercise, medication adherence	Regular text messages with lifestyle advice, monthly in-person education sessions	Monthly in-person education and health education booklet	5
Lison [18]	Spain	Outpatient clinic	18–65	Overweight or obese adults with diagnosis of hypertension (BP > 140/90 mmHg)	55	50	Diet, exercise	Access to online platform with modules for self-completion	Written lifestyle advice	3
Liu [33]	Canada	Online	35–74	Diagnosis of hypertension (BP > 140/90 mmHg if on no medications, or >130/85 if on medications)	100	97	Diet, exercise, smoking cessation, medication adherence	Regular emails with link to interactive e-counseling	Emailed regular written hypertension newsletter	3
Persell [31]	US	Community clinic	18–84	BP > 135/85 mmHg and <180/110 mmHg at screening visit	144	152	Diet, exercise, medication adherence, sleep, stress management	Home BPM and app providing reminders to check BP and lifestyle advice	Home BPM provided, standard care	6
Still [22]	US	Community clinic	>30	African-Americans with diagnosis of hypertension (BP > 140/90 mmHg)	30	30	Diet, exercise, medication adherence	Regular nurse telephone counselling, medication management app, home BPM, web-based education modules	Standard care, plus printed educational materials and access to one web-based session	3
Yun [20]	Korea	Outpatient clinic	>19	Diagnosis of hypertension	41	39	Diet, exercise, medication adherence	Access to app and web-based program encouraging lifestyle change	Health education booklet with healthy lifestyle habits	3
Kario [30]	Japan	Community clinic	20–64	Diagnosis of hypertension (BP 140–179/90–109 mmHg)	192	180	Diet, exercise, alcohol intake, sleep, stress	Home BPM and personalised lifestyle changes delivered via app	Written lifestyle advice provided	3

The pooled sample size for change in systolic blood pressure was 3040 participants (range 30 [32] to 493 participants [24]). Most studies included participants based on a previous diagnosis of hypertension [18, 20–23, 25, 26, 28–30, 33, 34], with some specifying this to be >140/90 mmHg [22, 33] or ≥140/90 mmHg [18, 22, 23, 25, 30, 33]. The rest included individuals based on screening visit blood pressure readings ≥135/85 mmHg [31], ≥140/90 mmHg [24, 27, 32], or >140/90 mmHg at a previous clinic visit [19].

Intervention duration ranged from 3 months to 12 months. Only two studies followed participants up beyond the end of the intervention. Both had interventions lasting 3 months but followed up their participants at 6 months [30] or 12 months [18]. The outcomes at the end of the intervention period were included in the meta-analysis, as either there was an option to add medication after the end of the intervention period [30], or later end-point values were not given for each group [18].

The nature of the digital intervention varied amongst studies (Table 2). Six studies used multi-faceted interventions that involved contact with a healthcare professional as well as a digital intervention (e.g. website access and nurse telephone counselling) [19, 21, 22, 26, 29, 34]. The remaining 11 studies had fully automated digital interventions, with no direct involvement from a healthcare professional [18, 20, 23–25, 27, 28, 30–33]. Eight studies had interventions that involved participants self-monitoring their blood pressure [19, 22, 24, 27, 30–32, 34].

**Table 2:** Intervention characteristics.

Study	Aspect of intervention	Intensity	Duration (months)	Person involvement?	Behavioural theories/techniques
Green [24]	Home blood pressure monitoring device with instructions to upload readings to website	Twice weekly	12	No	Nil mentioned
	Access to Group Health website, which includes facilities for refilling medication, viewing medical record, and resources for lifestyle change	Patient directed			
Bennett [21]	Motivational coaching sessions with dietician – two in person, two over the phone	Every 3 weeks	3	Yes	Motivational interviewing
	Website with self-monitoring of adherence to behaviour change goals	Patient directed			
	Session with health coach to select four obesogenic behaviour change goals	Once, at start of intervention			
Golshahi [23]	Regular SMS messages with self-management advice about medication adherence, increased physical activity, DASH diet, smoking cessation	Twice weekly	8	No	Nil mentioned
Kim [34]	Home blood pressure monitor, reminders to monitor and upload readings to app	Three times weekly	6	Yes	Nil mentioned
	iPhone with Healthy Circle app disease management platform with educational materials and behaviour recommendations. This could be accessed by healthcare professionals	Patient directed			
Liu [25]	Emails with links to website containing diet and exercise plans, information about setting behaviour goals and self-monitoring of lifestyle behaviours. Participants able to set their own goals or select interventions	Weekly	4	No	Transtheoretical model
Skolarus [27]	Home BP monitor and reminders to monitor and send readings to research team	Weekly	6	No	Self-determination theory
	Tailored text messaging in response to their BP measurements comparing most recent BP reading to enrolment BP	Weekly			
	Tailored healthy behaviour messages based on participant baseline characteristics	1–2 times weekly (patient choice)			
	Generic healthy behaviour advice for BP lowering	2–10 times weekly (patient choice)			
Li [26]	Health education and promotion articles related to hypertension, followed by a short quiz, sent via WeChat	1–2 times weekly (depending on risk group)	6	Yes	Self-efficacy theory
	Home blood pressure monitor and feedback on self-reported readings	Weekly			
	Access to WeChat group chats – able to discuss amongst participants and have private chat with researchers	Patient directed			
Meurer [32]	Home blood pressure monitor and reminders to text BP readings	Weekly	3	No	Self-determination theory
	Text messages with healthy behaviour lifestyle interventions	Weekly			
	Tailored text messages depending on whether participants took anti-hypertensive medication	Weekly			
Rehman [19]	Educational talk regarding lifestyle modifications during clinic visit	Once, at start of intervention	3	Yes	Nil mentioned
	Regular text messages with patient education for lifestyle changes	5 times weekly			
	Text messages with reminders to take medication	Twice daily			
	Home BP monitor and reminders to monitor	Weekly			
Borgstrom [28]	Text messages reminding patients to pursue healthy lifestyle change	Weekly	6	No	Nil mentioned
Jahan [29]	In-person health education provided by community health workers to encourage behaviour change, and health education booklet	Twice in the first month, then monthly	5	Yes	Nil mentioned
	Text messages with reminders for lifestyle change	5 times in the first month, then weekly			
Lison [18]	Webpage with online modules focused on obesity and hypertension	Weekly for first 5 weeks, then biweekly	3	No	Nil mentioned
	Downloadable documents and videos with lifestyle change advice	Patient directed			
Liu [33]	Emails with links to e-Counseling sessions to promote adherence to lifestyle change behaviours	Weekly for 4 months, then biweekly for 4 months, then monthly for 4 months	12	No	Prochaska’s transtheoretical model of health behaviour change
Persell [31]	Home blood pressure monitor and reminders to input readings	Daily for 3 weeks, then weekly	6	No	Cognitive behavioural therapy
	App with reminders and tracking of lifestyle change	Patient directed			
Still [22]	Web-based education modules with information about hypertension	Weekly for 6 weeks	3	Yes	Ryan and Swain’s individual and family self-management theory
	Home blood pressure monitor and tracking logs to record readings	Twice daily, twice weekly			
	Medication management app with SMS reminders to take medication	Patient directed			
	Nurse counselling sessions discussing medication adherence and BP monitoring	Once every 6 weeks			
Yun [20]	‘Smart Management Strategy for Health’ App and website, with materials to support health behaviour change and facility to create weekly plan for health management. Email reminders then sent based on their plan.	Weekly	3	No	12 rules for highly effective health behaviour and health management strategies
Kario [30]	Home blood pressure monitoring and app to upload readings. App then delivered personalised programme of lifestyle change advice	Patient directed	3	No	Nil mentioned

Control interventions varied between studies. Three studies had usual care as a comparator [23, 26, 28]. Ten studies had gave hypertension lifestyle advice in a ‘non-digital’ format, with eight providing written advice [18, 20, 21, 24, 27, 30–32] and two studies providing in-person hypertension education to control groups [19, 29]. Four studies gave control participants access to a limited element of the digital intervention received by the intervention group [22, 25, 33, 34].

11 studies had change in blood pressure as their primary outcome [19, 22–27, 30–33]. The remaining six had blood pressure measurement as secondary outcomes, with primary outcomes being change in BMI [18], change in weight [21], proportion of patients meeting pre-specified targets (glycated haemoglobin <7%, systolic blood pressure <140 mmHg, or low-density lipoprotein cholesterol <130 mg/dL) [20], study feasibility [28], and changes in health behaviour [29, 34].

Methods of blood pressure data collection included averages of repeat blood pressure measurements taken during study visits [18, 22, 24–26, 28, 31, 33, 34], self-reported home readings [19, 32], measurements taken in the community [27], 24-hour ambulatory blood pressure measurements [30], and was unspecified in 4 studies[20, 21, 23, 29].

### Risk of bias

**Fig. 2:**
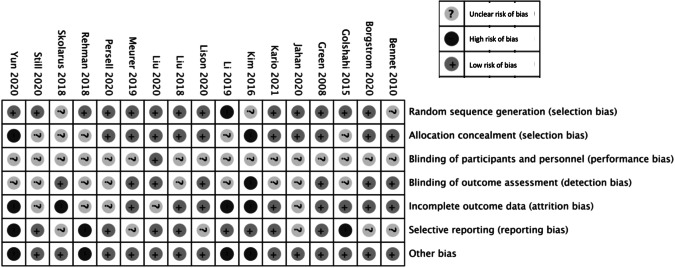
Risk of bias summary: review authors’ judgements about individual risk of bias domains for each included study.

Results from the risk of bias assessment are summarised in Fig. 2. Six studies had a high risk of bias in at least one domain [19, 20, 23, 26, 27, 34], and risk of bias was unclear in at least one domain for the remainder. Where high risk of bias was present, this was most commonly due to attrition bias. Of those studies included in the meta-analysis, three had a high risk of bias [23, 26, 27].

### Primary outcome

#### Systolic blood pressure

Eight studies performed statistical analysis comparing the change in systolic blood pressure between intervention and control groups [22, 24–27, 30, 31, 33], of which four found a significant reduction with the intervention [24, 26, 30, 33]. Three studies compared difference in systolic blood pressure change between multiple groups [23, 28, 29], with two finding a significant difference between groups [23, 29]. One study reported a significant difference in the proportion controlled <140/90 mmHg between groups [20]. The remaining studies did not compare blood pressure outcomes between groups.

**Fig. 3:**
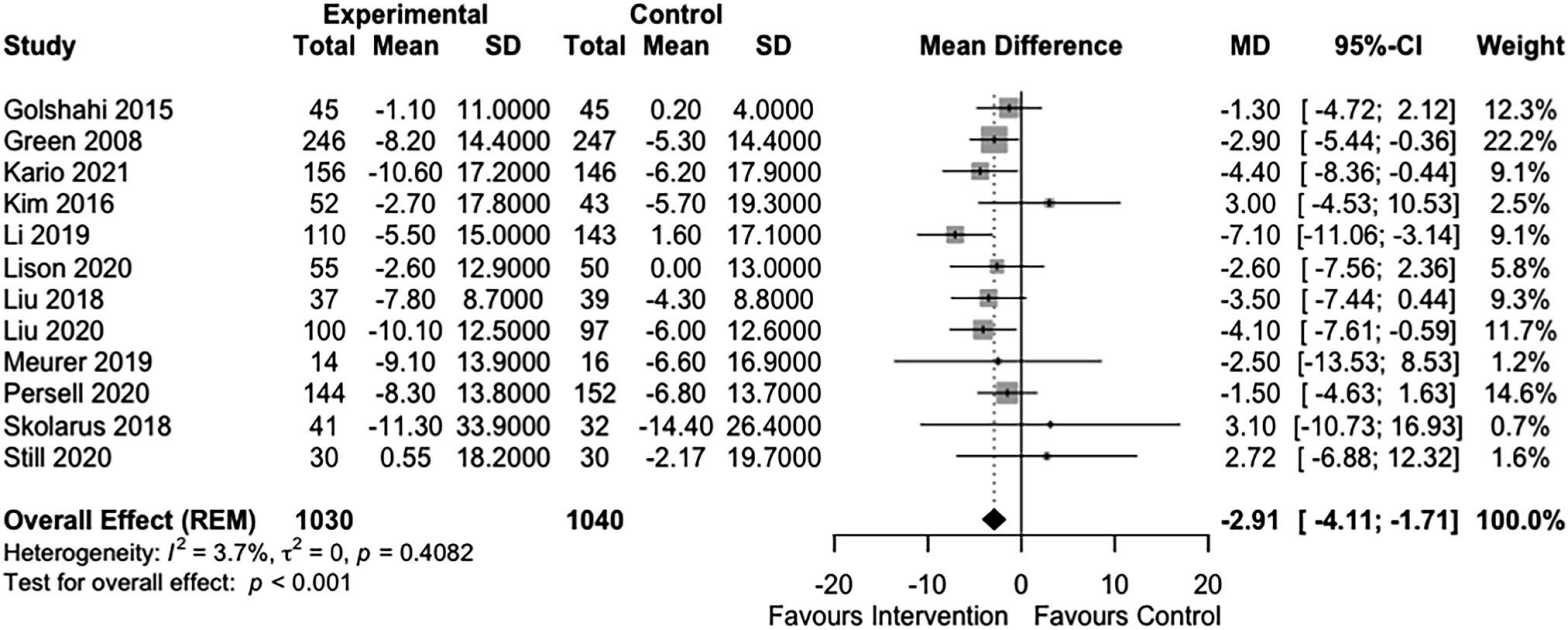
Forest plot of the effect of digital interventions targeting lifestyle factors for hypertension on systolic blood pressure.

Ten studies reported a mean change in systolic blood pressure from baseline to end point for both intervention and control groups [18, 23–27, 30–33] (See Supplementary Digital Content 2). Two further studies reported baseline and end point blood pressure values for both groups, from which a mean change and standard deviation was calculated [22, 34]. These 12 studies were included in the meta-analysis (Fig. 3). The remaining five reported insufficient numerical data for inclusion [19–21, 28, 29]. Blood pressure readings from study visits were reported and used by 10 of the studies [18, 22–27, 31, 33, 34]. The remaining two studies reported participant self-measured blood pressure, and these values were included in the meta-analysis [30, 32].

The forest plot in Fig. 3 summarises the effect of intervention on systolic blood pressure. Digital interventions were associated with a statistically significant reduction in systolic blood pressure compared to control (mean difference (MD) −2.91 mmHg; 95% confidence interval (CI) −4.11, −1.71; p value (p) <0.001; I^2^ = 4%, τ^2^ = 0).

#### Subgroup analysis

**Fig. 4:**
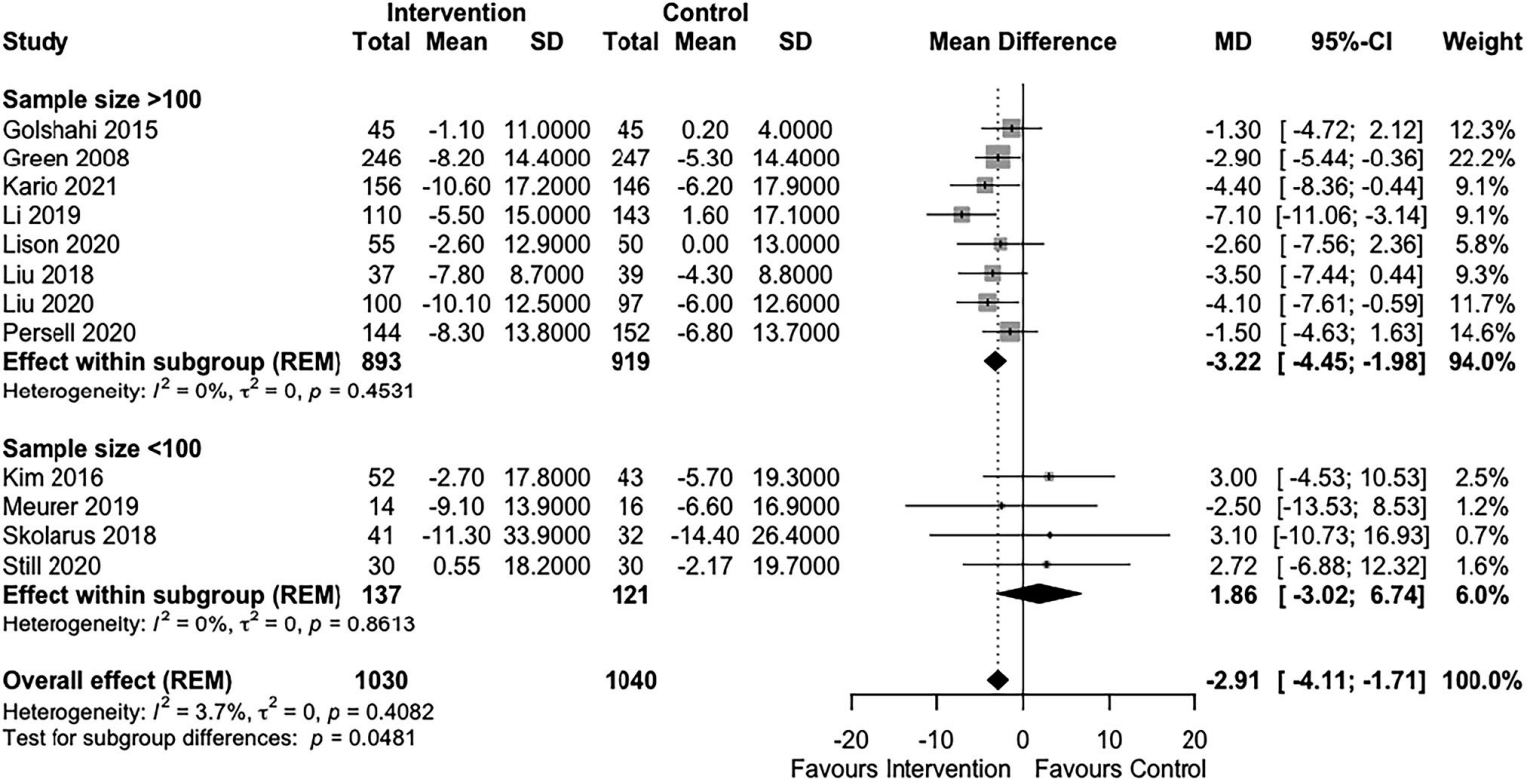
Subgroup analysis of effect of sample size on systolic blood pressure outcome.

Larger studies (sample size >100) had a greater effect size than smaller studies: mean SBP difference −3.22 mmHg (CI−4.45, −1.98) vs 1.86 mmHg (CI −3.02, 6.4) respectively, *p* < 0.05 (Fig. 4). No significant differences between subgroups were seen based on use of home blood pressure monitoring (*p* = 0.97), intervention duration (*p* = 0.91), nature of control group (*p* = 0.41) or clinician involvement (*p* = 0.7) (see Figures, Supplementary Digital Content 3–6).

#### Sensitivity analysis

A post-hoc sensitivity analysis was undertaken. A significant difference in change in systolic blood pressure was seen even when studies with unclear risk of bias in more than 2 domains, or high risk of bias in any domain were removed from analysis (−3.20 mmHg; CI −5.13, −1.26; *p* = 0.001) (see Figure, Supplementary Digital Content 7). A funnel plot of studies included in the meta-analysis did not suggest reporting bias in favour of the intervention. Most smaller studies did not show a blood pressure reducing effect (see Figure, Supplemental Digital Content 8).

#### Secondary outcomes

Ten studies included sufficient data for inclusion in a meta-analysis of diastolic blood pressure [18, 22–27, 30, 31, 34]. The mean difference in diastolic blood pressure change between groups was −1.13 mmHg (CI −1.91, −0.35), *p* = 0.005, I^2^ 0%, τ^2^ = < 0.0001 (see Figure, Supplementary Digital Content 9).

Reporting of other secondary outcomes was too heterogeneous between studies to allow meta-analysis (see Supplementary Digital Content 10). Three studies reported the change in BMI between groups [28, 30, 31], one of which found a statistically significant improvement in BMI with the intervention (−0.2 kg/m^2^, CI −.4 to −0.1, *p* = 0.005) [30]. Of three studies that reported values for weight change [21, 22, 30], only one performed an analysis to compare groups, and found a significant difference (−0.5 kg, *p* = 0.003) [30]. Four studies performed statistical analysis comparing physical activity measures between groups [20, 25, 31, 33], with one finding a significant difference in physical activity (*p* = 0.02 for difference in daily steps at 12 months) [33]. Reporting for salt intake (five studies [23, 29, 30, 33]), and/or dietary outcomes (five studies [23, 25, 29, 31, 33]) used entirely heterogenous outcomes. One study found a significant reduction in salt intake in the intervention group compared to control (*p* < 0.001), using check sheets filled by participants to measure salt intake. All studies reporting dietary outcomes found either no significant difference [25, 31, 33] or did not perform statistical analysis [23, 29]. Outcomes relating to smoking were only reported by two studies, each with a different measure [23, 34]. Neither compared between group changes in smoking measures.

## Discussion

This systematic review and meta-analysis finds a greater reduction in systolic blood pressure amongst hypertensive participants receiving a digital intervention to support lifestyle change, compared to controls. We hypothesise that the digital interventions lead to improved lifestyle modification, and thus mediate an improved blood pressure reduction. The intervention also proved effective for reduction in diastolic blood pressure. The actual mean systolic blood pressure reduction in the intervention group was −6.4 mmHg (compared to −4.6 mmHg in control group; between group difference of −2.91 mmHg). Previous studies suggest a blood pressure reduction of this size could lead to a risk reduction of 12% for coronary heart disease, and 15% for stroke [35]. However, interpretation of the results should bear in mind the limitations of the meta-analysis – the intervention, study duration and lifestyle factors targeted varied significantly between studies.

To our knowledge, this is the first meta-analysis of digital interventions in hypertension specifically targeting lifestyle factors to reduce blood pressure. The search was comprehensive (although the exclusion of articles not published in English is a limitation). There was no evidence of reporting bias suggested by our funnel plot. Instead, smaller studies showing a blood pressure reducing effect were missing. This may be due to smaller studies being underpowered to detect an effect, or due to inadequate methodology in smaller studies.

By excluding studies with interventions primarily focused on medication adherence, our results are particularly applicable to those individuals with hypertension using lifestyle change as a first step in management, prior to starting medication. This first step is recommended in many guidelines for low-risk individuals with modestly raised blood pressure [10]. Previous meta-analyses have assessed digital interventions aimed at medication adherence or titrating anti-hypertensives and have reported similar blood pressure reductions to those seen here [14].

Despite this study’s strengths, the interpretation of our results must consider a number of limitations. Most notably, there was significant heterogeneity across multiple components of the included studies, which particularly limits the interpretation of the meta-analysis. Studies varied in both the delivery method and intensity of the digital intervention, the lifestyle factors targeted, the method of blood pressure measurement, and the intervention duration. The meta-analysis groups these heterogeneous studies together, even though they are not all comparable across all variables. Recent meta-analyses of digital interventions targeting multiple aspects of hypertension in lower- and middle-income were similarly limited by heterogeneity between studies [36].

The inter-study variability was such that no consistent factor leading to blood pressure reduction could be identified in subgroup analysis by intervention type, and so we could not get insight into the mechanism by which a blood pressure reduction was mediated. The evidence base from which future digital interventions can be designed, or from which practitioners can guide their patients as to which apps to use, therefore remains limited, but worthy of further study. Future primary studies could be encouraged to report more clearly some of the above factors.

The treatment of control group participants was highly variable between studies (Table 1). Some studies used ‘usual care’ as a control, others delivered similar lifestyle advice to the intervention group but through non-digital means (e.g. education courses or printed information), whilst others received a digital intervention of differing intensity to the intervention group. Subgroup analysis by nature of control intervention showed a trend towards greater difference in blood pressure in studies where the intervention did not include any digital element, but the between subgroup difference was not significant (Supplementary Digital Content Fig. 5). Previous meta-analyses of the impact of non-digital interventions for lifestyle change in hypertension have shown similar blood pressure changes to those seen in this analysis [7]. It is possible that lifestyle modification facilitated by digital interventions provide at least a similar benefit to that delivered by non-digital means, whilst providing an advantage in terms of scalability and cost.

There remains some clinical uncertainty as to the benefit of digital approaches across different populations. Although our inclusion criteria selected for hypertensive populations, differences in individual study definitions of “hypertensive populations” resulted in a mean baseline blood pressure that was not consistently ≥140/90 mmHg (Table 1). There is increasing evidence that a reduction in blood pressure in individuals at risk of cardiovascular disease is beneficial regardless of the baseline blood pressure value [37], but it is difficult to predict the efficacy of a digital intervention in individuals with a systolic blood pressure ≥140 mmHg as few studies had mean baseline blood pressures in this range.

It should also be noted that the studies in this review were generally of short duration: only one study used an intervention for 12 months [24], with the intervention duration of the rest being 8 months or less. As a result, the longevity of the blood pressure reducing effect observed here remains unknown and future studies should include greater longitudinal follow up.

Although the conclusions of the review are tempered by the heterogeneity between studies, particularly in regard to the digital interventions used, this issue is not unique to digital interventions for lifestyle in hypertension – the American Heart Association found over 13,000 apps for cardiovascular disease in the Apple Store in 2015, few of which had undergone substantial testing for efficacy [38]. There is a growing consensus that these digital interventions need more rigorous testing [12, 38, 39]. We would support the same conclusion from our review.

In summary, lifestyle factor modification facilitated by digital interventions appears to lower blood pressure by small amounts compared with control interventions. However, heterogeneity in methodology and interventions used in the studies constrain the interpretation of the meta-analysis. As such, there is limited scope for making specific recommendations for clinical practice from this review. However, lifestyle interventions delivered digitally could offer rapidly scalable, low-cost and effective management options at a population level, potentially delaying or avoiding pharmacotherapy. Digital interventions in this context show promise but further research is required to understand the specific components which may be effective in blood pressure reduction before they can be integrated into existing care pathways.

## Summary

### What is known about the topic:

- Modification of lifestyle (weight loss, lowering salt intake, physical exercise, smoking cessation) can effectively lower blood pressure in individuals with hypertension, and is often recommended as a first management step.
- Digital interventions are being investigated in hypertension as they may offer a scalable, low-cost method to improve disease management.

### What this study adds:

- This study suggests digital interventions targeting lifestyle modification in hypertension result in a greater reduction in systolic and diastolic blood pressure than controls. Variability between the included studies limited understanding of the mechanism of blood pressure reduction.
- There is significant heterogeneity between available digital interventions, and further rigorous testing is required to identify the key factors that produce an effective blood pressure reduction.

## Supplementary information

Supplementary material

## Data Availability

All data included in this analysis have been taken from published articles and their supplementary files.
