# Supplementary material for: A systematic review and meta-analysis of digital interventions targeting lifestyle factors in patients with hypertension
Source: J Hum Hypertens. 2025 Aug 5;39(10):690–700. doi: 10.1038/s41371-025-01051-3 (PMC12500467; doi:10.1038/s41371-025-01051-3)
Supplement: Supplementary file 1 — Supplementary material [file 41371_2025_1051_MOESM1_ESM.pdf]

Supplementary digital content

SDC 1. Search Strategy

SDC 2. Summary of blood pressure outcome reporting in included studies

SDC 3. Subgroup analysis of effect of home blood pressure monitoring on systolic blood pressure outcome

SDC 4. Subgroup analysis of effect of intervention duration on systolic blood pressure outcome

SDC 5. Subgroup analysis of effect of control characteristics on systolic blood pressure outcome

SDC 6. Subgroup analysis of effect of person involvement on systolic blood pressure outcome

SDC 7. Sensitivity analysis of effect of bias risk on systolic blood pressure outcome

SDC 8. Funnel plot of studies included in the meta-analysis

SDC 9. Forest plot of the effect on diastolic blood pressure of digital interventions targeting lifestyle factors for hypertension

SDC 10. Summary of secondary outcomes reporting in included studies

SDC 1. Search Strategy

MEDLINE (Ovid) search strategy

1. hypertension/ or essential hypertension/
2. (blood pressure or hypertens*).ti,ab.
3. 1 or 2 or 3
4. cell phone/ or text messaging/
5. software/ or mobile applications/ or user-computer interface/ or web browser/
6. computers, handheld/ or smartphone/
7. Videoconferencing/
8. exp Internet/
9. Social Media/
10. electronic mail/ or telemedicine/ or remote consultation/ or telepathology/ or telerehabilitation/
11. social networking/ or online social networking/
12. wearable electronic devices/ or fitness trackers/
13. (text messag* or sms or short messag* service*).ti,ab.

14. (telephone or phone or mobile phone or cell phone or cellphone or smartphone or smart phone or i-phone or iphone or i-pad or ipad or app or apps or mobile application*).ti,ab.

15. (mobile health or mobile technolog* or mhealth).ti,ab.

16. (email or e-mail or electronic mail or web* or website* or web site* or internet or online).ti,ab.

17. ((digital or electronic or technol* or computer) adj5 (based or intervention or deliver*)).ti,ab.

18. (laptop or computer).ti,ab.

19. (wearable adj3 (device or technolog* or monitor or tracker or sensor)).ti,ab.

20. (fitbit or fit bit or pedometer or accelerometer or fitness tracker).ti,ab.

21. exp Exercise/

22. exercise therapy/ or endurance training/ or plyometric exercise/ or resistance training/

23. exp Diet Therapy/

24. Nutrition Therapy/

25. exp Drinking Behavior/

26. Diet, Sodium-Restricted/

27. sodium, dietary/ or sodium chloride, dietary/

28. health behavior/ or smoking cessation/ or smoking reduction/ or "tobacco use cessation"/

29. health promotion/ or healthy people programs/ or weight reduction programs/

30. risk reduction behavior/

31. Preventive Medicine/

32. (exercise or physical activity or sport* or walk* or running or jog* or swimming or cycling).ti,ab.

33. Patient Education as Topic/

34. (exercise* adj3 (intervention or therapy or program* or train*)).ti,ab.

35. (physical adj3 (endurance or education or fitness or activit* or train*)).ti,ab.

36. ((diet* or food) adj3 (intervention* or modif*)).ti,ab.

37. (health* adj2 eating).ti,ab.

38. dieting.ti,ab.

39. (nutrition* adj3 (counselling or counseling or therapy)).ti,ab.

40. (weight train* or weight reduction or weight loss program*).ti,ab.

41. (weight adj3 (loss or reduc* or decreas* or change* or control* or manage*)).ti,ab.

42. ((BMI or body mass index) adj3 (loss or reduc* or decreas* or change* or control*)).ti,ab.

43. ((alcohol or drink) adj3 (reduc* or decrease* or chang*)).ti,ab.

44. alcohol.ti.

45. ((salt or sodium) adj3 (low or reduc* or restrict or chang* or decrease or diet*)).ti,ab.

46. (health* adj3 behavio?r*).ti,ab.

47. ((behavio?r* or lifestyle) adj3 (chang* or modif* or therapy or therapies or program* or intervention* or counsel* or advice)).ti,ab.

48. (self-management or self management).ti,ab.

49. ((cigarette* or smoking or tobacco) adj3 (reduc* or quit* or cessation or decreas*)).ti,ab.

50. 5 or 6 or 7 or 8 or 9 or 10 or 11 or 12 or 13 or 14 or 15 or 16 or 17 or 18 or 19 or 20 or 21

51. 22 or 23 or 24 or 25 or 26 or 27 or 28 or 29 or 30 or 31 or 32 or 33 or 34 or 35 or 36 or 37 or 38 or 39 or 40 or 41 or 42 or 43 or 44 or 45 or 46 or 47 or 48 or 49 or 50

52. randomized controlled trial.pt.

53. controlled clinical trial.pt.

54. randomized.ab.

55. placebo.ab.

56. drug therapy.fs.

57. randomly.ab.

58. groups.ab.

59. (trial or multicenter or multi center or multicentre or multi centre).ti,ab.

60. multicenter study.pt.

61. pragmatic clinical trial.pt.

62. (intervention? or effect? or impact? or controlled or control group? or (before adj5 after) or (pre adj5 post) or ((pretest or pre test) and (posttest or post test)) or quasiexperiment* or quasi experiment* or evaluat* or time series or time point? or repeated measur* or ((feasibility or pilot) adj (study or project*))).ti,ab.

63. 53 or 54 or 55 or 56 or 57 or 58 or 59 or 60 or 61 or 62 or 63

64. exp animals/ not humans/

65. 64 not 65

66. 4 and 51 and 52 and 66

67. limit 67 to yr="2000 -Current"

Embase search strategy

1. blood pressure/ or arterial pressure/ or blood pressure fluctuation/ or blood pressure regulation/ or diastolic blood pressure/ or systolic blood pressure/

2. hypertension/ or elevated blood pressure/ or borderline hypertension/ or essential hypertension/ or prehypertension/ or systolic hypertension/

3. (blood pressure or hypertens*).ti,ab.
4. 1 or 2 or 3
5. mobile phone/ or smartphone/
6. exp mobile application/
7. text messaging/
8. web browser/
9. computer interface/ or information technology device/
10. tablet computer/
11. computer assisted therapy/
12. telephone/
13. smartphone/
14. videoconferencing/
15. exp internet/
16. e-mail/
17. telemedicine/ or telehealth/ or teleconsultation/ or telemonitoring/ or telepathology/ or telerehabilitation/

18. social media/

19. blogging/

20. webcast/

21. electronic device/

22. (text messag* or sms or short messag* service*).ti,ab.

23. (telephone or phone or mobile phone or cell phone or cellphone or smartphone or smart phone or i-phone or iphone or i-pad or ipad or app or apps or mobile application*).ti,ab.

24. (mobile health or mobile technolog* or mhealth).ti,ab.

25. (email or e-mail or electronic mail or web* or website* or web site* or internet or online).ti,ab.

26. ((digital or electronic or technol* or computer) adj5 (based or intervention or deliver*)).ti,ab.

27. (laptop or computer).ti,ab.

28. (wearable adj3 (device or technolog* or monitor or tracker or sensor)).ti,ab.

29. (fitbit or fit bit or pedometer or accelerometer or fitness tracker).ti,ab.

30. exp exercise/

31. kinesiotherapy/

32. exp diet/

33. diet therapy/ or dash diet/ or diabetic diet/ or diet restriction/ or low calorie diet/ or low fat diet/ or low glycemic index diet/

34. alcohol consumption/

35. drinking behavior/

36. sodium restriction/

37. exp health behavior/

38. smoking cessation/ or smoking cessation program/

39. health promotion/

40. exp body weight loss/

41. exp risk reduction/

42. preventive medicine/

43. exp patient education/

44. (exercise or physical activity or sport* or walk* or running or jog* or swimming or cycling).ti,ab.

45. (exercise* adj3 (intervention or therapy or program* or train*)).ti,ab.

46. (physical adj3 (endurance or education or fitness or activit* or train*)).ti,ab.

47. ((diet* or food) adj3 (intervention* or modif*)).ti,ab.

48. (health* adj2 eating).ti,ab.

49. dieting.ti,ab.

50. (nutrition* adj3 (counselling or counseling or therapy)).ti,ab.

51. (weight train* or weight reduction or weight loss program*).ti,ab.

52. (weight adj3 (loss or reduc* or decreas* or change* or control* or manage*)).ti,ab.

53. ((BMI or body mass index) adj3 (loss or reduc* or decreas* or change* or control*)).ti,ab.

54. ((alcohol or drink) adj3 (reduc* or decrease* or chang*)).ti,ab.

55. alcohol.ti.

56. ((salt or sodium) adj3 (low or reduc* or restrict or chang* or decrease or diet*)).ti,ab.

57. (health* adj3 behavio?r*).ti,ab.

58. ((behavio?r* or lifestyle) adj3 (chang* or modif* or therapy or therapies or program* or intervention* or counsel* or advice)).ti,ab.

59. (self-management or self management).ti,ab.

60. 5 or 6 or 7 or 8 or 9 or 10 or 11 or 12 or 13 or 14 or 15 or 16 or 17 or 18 or 19 or 20 or 21 or 22 or 23 or 24 or 25 or 26 or 27 or 28 or 29

61. 30 or 31 or 32 or 33 or 34 or 35 or 36 or 37 or 38 or 39 or 40 or 41 or 42 or 43 or 44 or 45 or 46 or 47 or 48 or 49 or 50 or 51 or 52 or 53 or 54 or 55 or 56 or 57 or 58 or 59

62. exp controlled clinical trial/ or pretest posttest control group design/

63. single blind procedure/ or double blind procedure/

64. crossover procedure/

65. intervention study/

66. multicenter study/

67. (random or ((singl* or doubl*) adj (blind* or mask*)) or crossover or cross over or factorial* or latin square or assign* or allocat* or volunteer*).ti,ab.

68. (trial or multicenter or multi center or multicentre or multi centre).ti,ab.

69. (intervention? or effect? or impact? or controlled or control group? or (before adj5 after) or (pre adj5 post) or ((pretest or pre test) and (posttest or post test)) or quasiexperiment* or quasi experiment* or evaluat* or time series or time point? or repeated measur* or ((feasibility or pilot) adj (study or project*))).ti,ab.

70. 62 or 63 or 64 or 65 or 66 or 67 or 68 or 69

71. (exp animals/ or nonhuman/) not human/

72. 70 not 71

73. 4 and 60 and 61 and 72

74. limit 73 to yr="2000 -Current"

75. limit 74 to conference abstracts

76. 74 not 75

CENTRAL search strategy

1. MeSH descriptor: [Blood Pressure] explode all trees
2. MeSH descriptor: [Hypertension] this term only
3. MeSH descriptor: [Essential Hypertension] explode all trees
4. MeSH descriptor: [Prehypertension] explode all trees
5. #1 or #2 or #3 or #4
6. MeSH descriptor: [Cell Phone] explode all trees
7. MeSH descriptor: [Cell Phone Use] this term only
8. MeSH descriptor: [Smartphone] explode all trees
9. MeSH descriptor: [Mobile Applications] this term only
10. MeSH descriptor: [User-Computer Interface] this term only

11. MeSH descriptor: [Web Browser] this term only
12. MeSH descriptor: [Computers, Handheld] this term only
13. MeSH descriptor: [Videoconferencing] this term only
14. MeSH descriptor: [Internet] explode all trees
15. MeSH descriptor: [Social Media] this term only
16. MeSH descriptor: [Text Messaging] this term only
17. MeSH descriptor: [Electronic Mail] this term only
18. MeSH descriptor: [Social Networking] this term only
19. MeSH descriptor: [Online Social Networking] this term only
20. MeSH descriptor: [Wearable Electronic Devices] this term only
21. MeSH descriptor: [Fitness Trackers] this term only
22. ((text messag* or sms or short messag* service*)):ti,ab,kw OR ((telephone or phone or mobile phone or cell phone or cellphone or smartphone or smart phone or i-phone or iphone or i-pad or ipad or app or apps or mobile application*)):ti,ab,kw OR ((mobile health or mobile technolog* or mhealth)):ti,ab,kw OR ((email or e-mail or electronic mail or web* or website* or web site* or

internet or online)):ti,ab,kw OR (((digital or electronic or technol* or computer)NEAR/5(based or intervention or deliver*))):ti,ab,kw

23. (laptop or computer):ti,ab,kw OR ((wearable)NEAR/3(device or technolog* or monitor or tracker or sensor)):ti,ab,kw OR (fitbit or fit bit or pedometer or accelerometer or fitness tracker):ti,ab,kw
24. #6 or #7 or #8 or #9 or #10 or #11 or #12 or #13 or #14 or #15 or #16 or #17 or #18 or #19 or #20 or #21 or #22 or #23
25. MeSH descriptor: [Exercise] explode all trees
26. MeSH descriptor: [Exercise Therapy] explode all trees
27. MeSH descriptor: [Diet Therapy] explode all trees
28. MeSH descriptor: [Nutrition Therapy] this term only
29. MeSH descriptor: [Drinking Behavior] explode all trees
30. MeSH descriptor: [Diet, Sodium-Restricted] this term only
31. MeSH descriptor: [Sodium, Dietary] this term only
32. MeSH descriptor: [Sodium Chloride, Dietary] this term only
33. MeSH descriptor: [Health Behavior] explode all trees
34. MeSH descriptor: [Health Promotion] explode all trees

35. MeSH descriptor: [Risk Reduction Behavior] this term only

36. MeSH descriptor: [Preventive Medicine] this term only

37. MeSH descriptor: [Patient Education as Topic] explode all trees

38. ((exercise or physical activity or sport* or walk* or running or jog* or swimming or cycling)):ti,ab,kw OR (((exercise*)NEAR/3(intervention or therapy or program* or train*))):ti,ab,kw OR (((physical)NEAR/3(endurance or education or fitness or activit* or train*))):ti,ab,kw OR (((diet* or food)NEAR/3(intervention* or modif*))):ti,ab,kw OR ((health*)NEAR/2(eating)):ti,ab,kw

39. (dieting):ti,ab,kw OR ((nutrition*)NEAR/3(counselling or counseling or therapy)):ti,ab,kw OR (weight train* or weight reduction or weight loss program*):ti,ab,kw OR ((weight)NEAR/3(loss or reduc* or decreas* or change* or control* or manage*)):ti,ab,kw OR ((BMI or body mass index)NEAR/3(loss or reduc* or decreas* or change* or control*)):ti,ab,kw

40. ((alcohol or drink)NEAR/3(reduc* or decrease* or chang*)):ti,ab,kw OR (alcohol):ti OR ((salt or sodium)NEAR/3(low or reduc* or restrict or chang* or decrease or diet*)):ti,ab,kw OR ((health*)NEAR/3(behavio?r*)):ti,ab,kw OR ((behavio?r* or lifestyle)NEAR/3(chang* or modif* or therapy or therapies or program* or intervention* or counsel* or advice)):ti,ab,kw

41. ((self-management or self management)):ti,ab,kw OR ((cigarette* or smoking or tobacco)NEAR/3(reduc* or quit* or cessation or decreas*)):ti,ab,kw

42. #25 or #26 or #27 or #28 or #29# or #30 or #31 or #32 or #33 or #34 or #35 or #36 or #37 or #38 or #39 or #40 or #41

43. #5 and #24 and #42 with Publication Year from 2000 to 2020, in Trial

SDC 2. Summary of blood pressure outcome reporting in included studies

Study	n		Baseline SBP (SD) mmHg		Primary outcome	Reported BP values	p value comparison	p value
	Int	Cont	Int	Cont				
Green, 2008**[21]	246	247	152.2 (10)	151.3 (10.6)	Change in blood pressure	Mean SBP change from baseline to end point for both groups	Difference in SBP change between groups	0 02
Bennett, 2010[18]	51	50	134.7 (16.9)	137.1 (13.7)	Change in weight	Mean difference in SBP change between groups	No p value for between group difference	
Golshahi, 2015*,**[33]	45	45	149.6 (12.6)	149.6 (14.3)	Change in blood pressure	Mean SBP at baseline and endpoint for both groups	ANOVA for between group differences	<0.001
Kim, 2016*[24]	52	43	136.1 (15.2)	145.9 (19.5)	Change in medication adherence score	Mean SBP at baseline and endpoint for both groups	No p value for between group difference	
Liu, 2018*[26]	37	39	140.0 (11.6)	138.6 (11.2)	Change in systolic blood pressure	Mean SBP change from baseline to end point for both groups	Difference in SBP change between groups	0 14
Skolarus, 2018* [22]	41	32	160.7 (23.6)	162.2 (20.5)	Change in blood pressure	Mean SBP change from baseline to end point for both groups	Difference in SBP change between groups	0 6
Li, 2019* [31]	110	143	135.8 (15.9)	135.2 (14.8)	Change in systolic blood pressure	Mean difference in SBP change between groups	Difference in SBP change between groups	<0.001
Meurer, 2019* [23]	14	16	Not reported	Not reported	Proportion of patients with persistent hypertension	Mean SBP change from baseline to end point for both groups	No p value for between group difference	
Rehman, 2019[30]	Not specified	Not specified	149.3 (5.6)	148.9 (5.5)	Change in blood pressure	Mean SBP at baseline and endpoint for both groups	No p value for between group difference	
Borgstrom, 2020[27]	29	28	141.7 (13.8)	141.1 (13.8)	Feasibility of study	Mean difference in SBP change between groups	ANCOVA for between group differences	0 19
Jahan, 2020[29]	204	208	136.9 (19.2)	136.9 (19.3)	Evaluation of self-reported lifestyle change	Mean SBP at baseline and endpoint for both groups	ANOVA for between group differences	0 04
Lison, 2020*[28]	55	50	132.2 (14.2)	128.5 (13.5)	Change in BMI	Mean SBP change from baseline to end point for both groups	No p value for between group difference	

Liu, 2020* [25]	100	97	141.5 (12.2)	140.6 (12.6)	Change in blood pressure	Mean difference in SBP change between groups	Difference in SBP change between groups	0 02
Persell, 2020* [20]	144	152	140.6 (12.2)	141.8 (13.4)	Change in SBP	Mean SBP change from baseline to end point for both groups	Difference in SBP change between groups	0 16
Still, 2020*[19]	30	30	138.5 (14.8)	141.2 (16.9)	Change in blood pressure	Mean SBP change from baseline to end point for both groups	Difference in SBP change between groups	0 89
Yun, 2020[32]	41	39	Not reported	Not reported	Percentage of patients meeting clinical target indicators	No blood pressure values given	Difference in percentage of patients achieving BP target <140/90mmHg	0 04
Kario, 2021*s[34]	192	180	144.9 (10.4)	144.3 (10.4)	Change in 24hour ambulatory BP	Mean SBP change from baseline to end point for both groups	Difference in SBP change between groups (morning home SBP)	<0.001

**=study included in SBP meta-analysis, **=study with >2 groups*

SDC 3. Subgroup analysis of effect of home blood pressure monitoring on systolic blood pressure outcome

Study	Intervention Total	Intervention Mean	Intervention SD	Control Total	Control Mean	Control SD	MD	95%-CI	Weight
**Home BP Monitor**									
Green 2008	246	-8.20	14.4000	247	-5.30	14.4000	-2.90	[-5.44; -0.36]	22.2%
Kario 2021	156	-10.60	17.2000	146	-6.20	17.9000	-4.40	[-8.36; -0.44]	9.1%
Kim 2016	52	-2.70	17.8000	43	-5.70	19.3000	3.00	[-4.53; 10.53]	2.5%
Li 2019	110	-5.50	15.0000	143	1.60	17.1000	-7.10	[-11.06; -3.14]	9.1%
Meurer 2019	14	-9.10	13.9000	16	-6.60	16.9000	-2.50	[-13.53; 8.53]	1.2%
Persell 2020	144	-8.30	13.8000	152	-6.80	13.7000	-1.50	[-4.63; 1.63]	14.6%
Skolarus 2018	41	-11.30	33.9000	32	-14.40	26.4000	3.10	[-10.73; 16.93]	0.7%
Still 2020	30	0.55	18.2000	30	-2.17	19.7000	2.72	[-6.88; 12.32]	1.6%
**Effect within subgroup (REM)**	**793**			**809**			**-2.79**	**[-4.89; -0.70]**	**61.0%**
Heterogeneity: $I^2 = 30.2\%$, $\tau^2 = 2.5638$, $p = 0.1869$									
**No Home BP Monitor**									
Golshahi 2015	45	-1.10	11.0000	45	0.20	4.0000	-1.30	[-4.72; 2.12]	12.3%
Lison 2020	55	-2.60	12.9000	50	0.00	13.0000	-2.60	[-7.56; 2.36]	5.8%
Liu 2018	37	-7.80	8.7000	39	-4.30	8.8000	-3.50	[-7.44; 0.44]	9.3%
Liu 2020	100	-10.10	12.5000	97	-6.00	12.6000	-4.10	[-7.61; -0.59]	11.7%
**Effect within subgroup (REM)**	**237**			**231**			**-2.85**	**[-4.77; -0.94]**	**39.0%**
Heterogeneity: $I^2 = 0\%$, $\tau^2 = 0$, $p = 0.7074$									
**Overall effect (REM)**	**1030**			**1040**			**-2.91**	**[-4.11; -1.71]**	**100.0%**
Heterogeneity: $I^2 = 3.7\%$, $\tau^2 = 0$, $p = 0.4082$									
Test for subgroup differences: $p = 0.9655$									

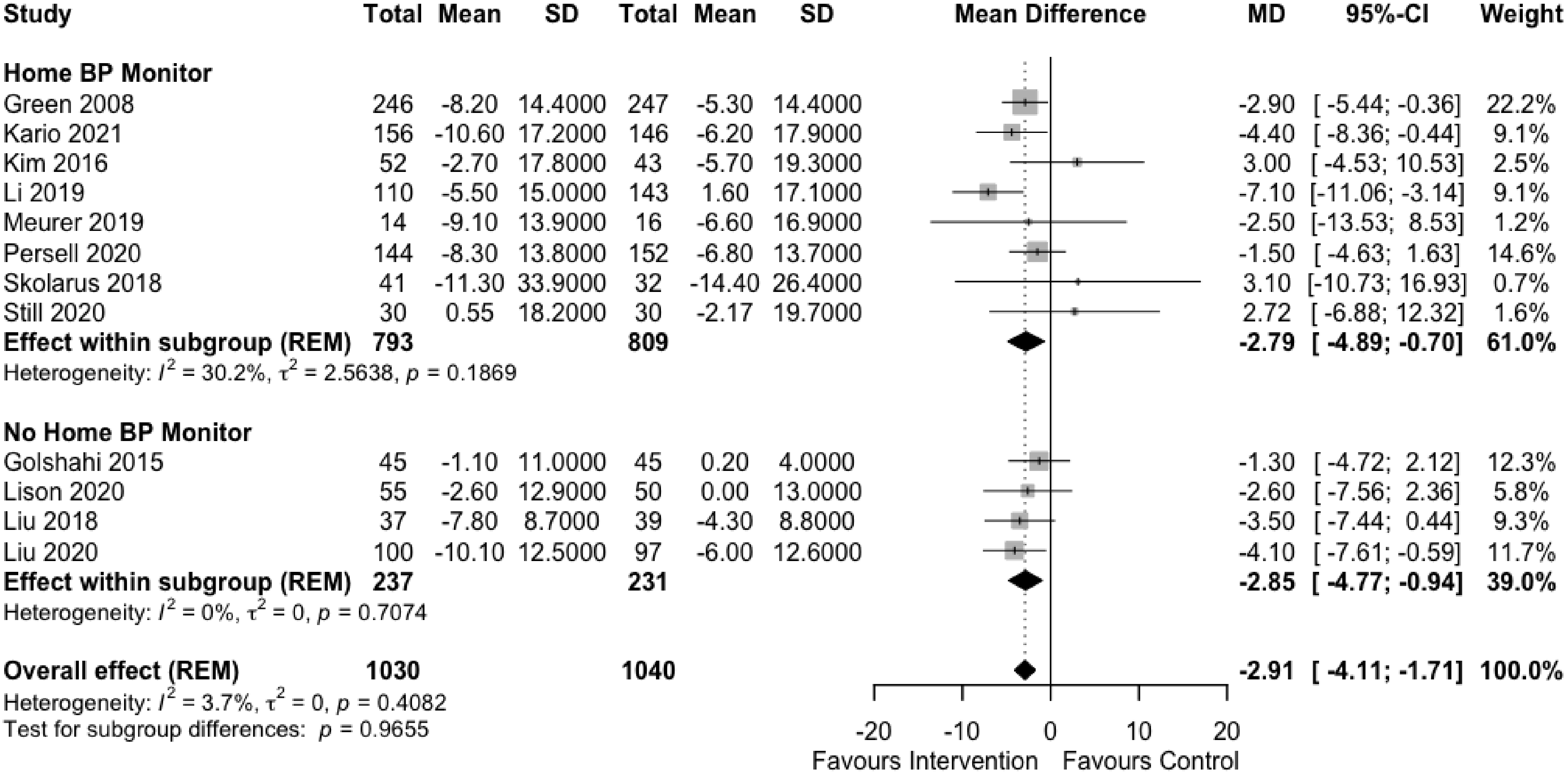

SDC 4. Subgroup analysis of effect of intervention duration on systolic blood pressure outcome

	Intervention			Control					
**Study**	**Total**	**Mean**	**SD**	**Total**	**Mean**	**SD**	**MD**	**95%-CI**	**Weight**
**Intervention Duration = 3 months or less**									
Golshahi 2015	45	-1.10	11.0000	45	0.20	4.0000	-1.30	[-4.72; 2.12]	12.3%
Green 2008	246	-8.20	14.4000	247	-5.30	14.4000	-2.90	[-5.44; -0.36]	22.2%
Kim 2016	52	-2.70	17.8000	43	-5.70	19.3000	3.00	[-4.53; 10.53]	2.5%
Li 2019	110	-5.50	15.0000	143	1.60	17.1000	-7.10	[-11.06; -3.14]	9.1%
Liu 2018	37	-7.80	8.7000	39	-4.30	8.8000	-3.50	[-7.44; 0.44]	9.3%
Liu 2020	100	-10.10	12.5000	97	-6.00	12.6000	-4.10	[-7.61; -0.59]	11.7%
Persell 2020	144	-8.30	13.8000	152	-6.80	13.7000	-1.50	[-4.63; 1.63]	14.6%
Skolarus 2018	41	-11.30	33.9000	32	-14.40	26.4000	3.10	[-10.73; 16.93]	0.7%
**Effect within subgroup (REM)**	**775**			**798**			**-2.88**	**[-4.30; -1.46]**	**82.3%**
Heterogeneity: $I^2 = 26.6\%$, $\tau^2 = 0.4319$, $p = 0.2161$									
**Intervention Duration = More than 3 months**									
Kario 2021	156	-10.60	17.2000	146	-6.20	17.9000	-4.40	[-8.36; -0.44]	9.1%
Lison 2020	55	-2.60	12.9000	50	0.00	13.0000	-2.60	[-7.56; 2.36]	5.8%
Meurer 2019	14	-9.10	13.9000	16	-6.60	16.9000	-2.50	[-13.53; 8.53]	1.2%
Still 2020	30	0.55	18.2000	30	-2.17	19.7000	2.72	[-6.88; 12.32]	1.6%
**Effect within subgroup (REM)**	**255**			**242**			**-3.05**	**[-5.90; -0.21]**	**17.7%**
Heterogeneity: $I^2 = 0\%$, $\tau^2 = 0$, $p = 0.5987$									
**Overall effect (REM)**	**1030**			**1040**			**-2.91**	**[-4.11; -1.71]**	**100.0%**
Heterogeneity: $I^2 = 3.7\%$, $\tau^2 = 0$, $p = 0.4082$									
Test for subgroup differences: $p = 0.9136$									

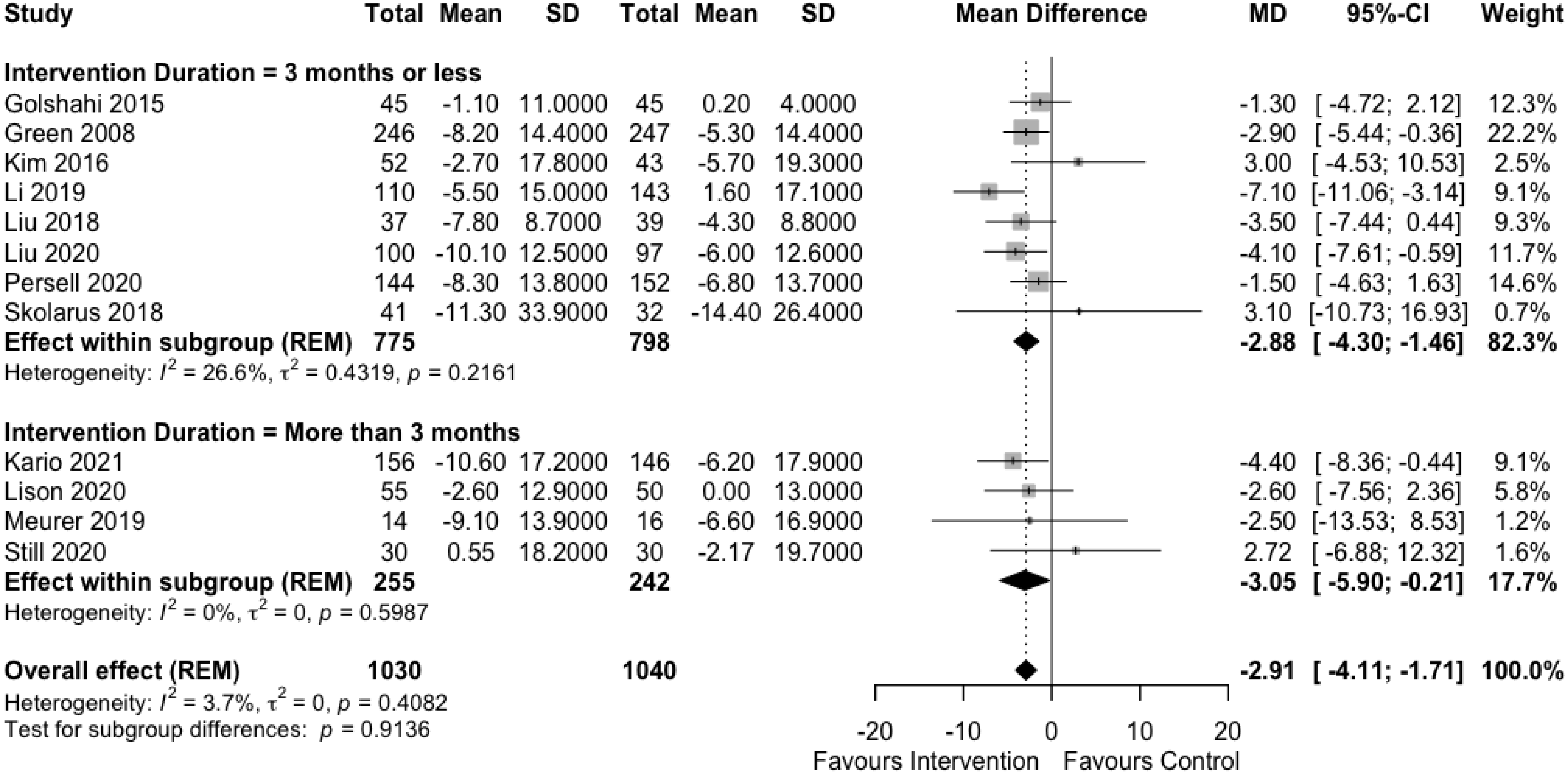

SDC 5. Subgroup analysis of effect of control characteristics on systolic blood pressure outcome

Study	Intervention Total	Intervention Mean	Intervention SD	Control Total	Control Mean	Control SD	MD	95%-CI	Weight
**Digital Control**									
Kim 2016	52	-2.70	17.8000	43	-5.70	19.3000	3.00	[-4.53; 10.53]	2.5%
Liu 2018	37	-7.80	8.7000	39	-4.30	8.8000	-3.50	[-7.44; 0.44]	9.3%
Liu 2020	100	-10.10	12.5000	97	-6.00	12.6000	-4.10	[-7.61; -0.59]	11.7%
Meurer 2019	14	-9.10	13.9000	16	-6.60	16.9000	-2.50	[-13.53; 8.53]	1.2%
Persell 2020	144	-8.30	13.8000	152	-6.80	13.7000	-1.50	[-4.63; 1.63]	14.6%
Still 2020	30	0.55	18.2000	30	-2.17	19.7000	2.72	[-6.88; 12.32]	1.6%
**Effect within subgroup (REM)**	**377**			**377**			**-2.29**	**[-4.16; -0.41]**	**40.8%**
Heterogeneity: $I^2 = 0\%$, $\tau^2 = 0$, $p = 0.4698$									
**Non-digital Control**									
Golshahi 2015	45	-1.10	11.0000	45	0.20	4.0000	-1.30	[-4.72; 2.12]	12.3%
Green 2008	246	-8.20	14.4000	247	-5.30	14.4000	-2.90	[-5.44; -0.36]	22.2%
Kario 2021	156	-10.60	17.2000	146	-6.20	17.9000	-4.40	[-8.36; -0.44]	9.1%
Li 2019	110	-5.50	15.0000	143	1.60	17.1000	-7.10	[-11.06; -3.14]	9.1%
Lison 2020	55	-2.60	12.9000	50	0.00	13.0000	-2.60	[-7.56; 2.36]	5.8%
Skolarus 2018	41	-11.30	33.9000	32	-14.40	26.4000	3.10	[-10.73; 16.93]	0.7%
**Effect within subgroup (REM)**	**653**			**663**			**-3.38**	**[-5.17; -1.59]**	**59.2%**
Heterogeneity: $I^2 = 18.4\%$, $\tau^2 = 0.9088$, $p = 0.2937$									
**Overall effect (REM)**	**1030**			**1040**			**-2.91**	**[-4.11; -1.71]**	**100.0%**
Heterogeneity: $I^2 = 3.7\%$, $\tau^2 = 0$, $p = 0.4082$									
Test for subgroup differences: $p = 0.4078$									

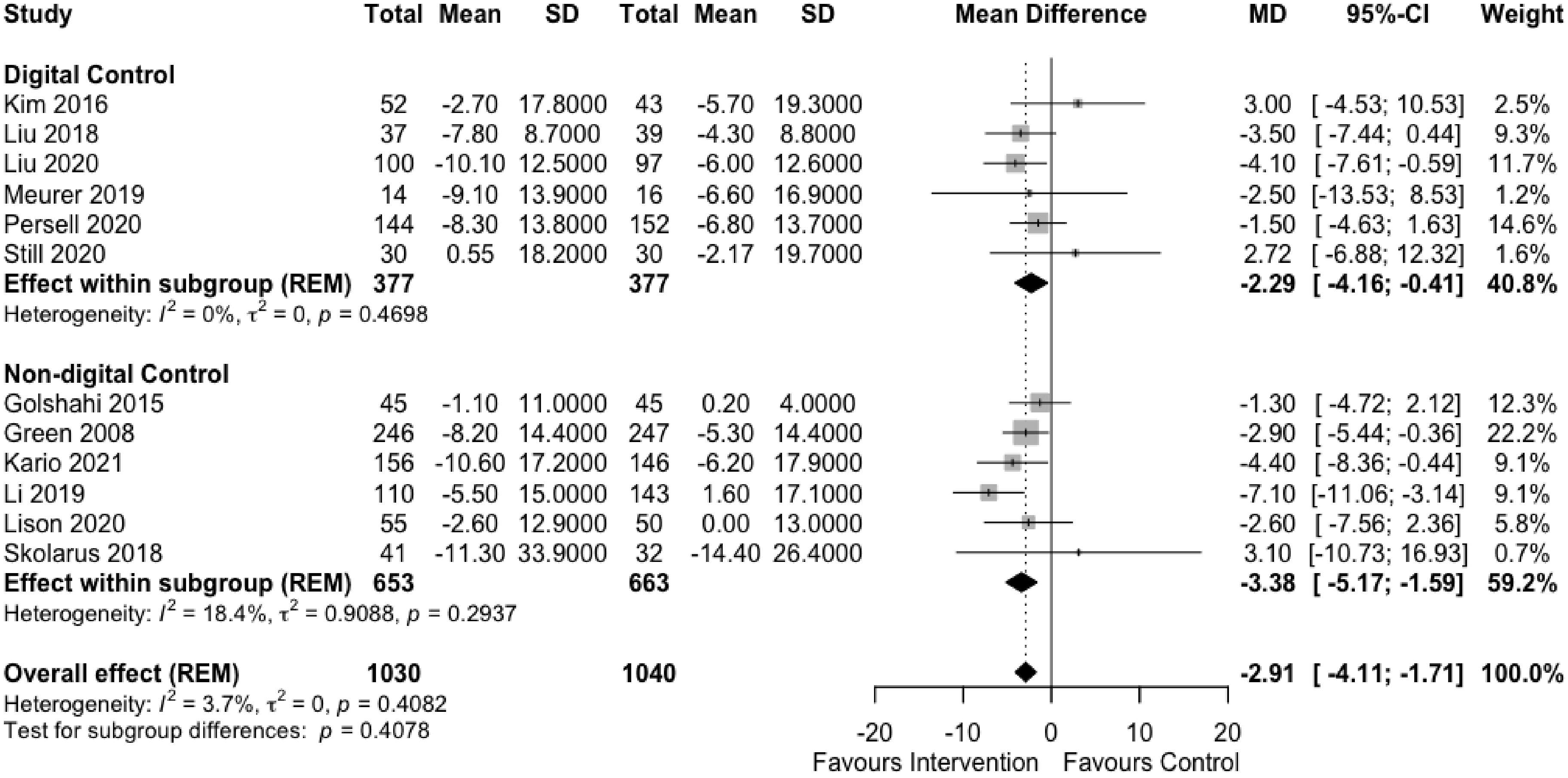

SDC 6. Subgroup analysis of effect of person involvement on systolic blood pressure outcome

Study	Intervention Total	Intervention Mean	Intervention SD	Control Total	Control Mean	Control SD	MD	95%-CI	Weight
**No Person Involvement**									
Golshahi 2015	45	-1.10	11.0000	45	0.20	4.0000	-1.30	[-4.72; 2.12]	12.3%
Green 2008	246	-8.20	14.4000	247	-5.30	14.4000	-2.90	[-5.44; -0.36]	22.2%
Kario 2021	156	-10.60	17.2000	146	-6.20	17.9000	-4.40	[-8.36; -0.44]	9.1%
Lison 2020	55	-2.60	12.9000	50	0.00	13.0000	-2.60	[-7.56; 2.36]	5.8%
Liu 2018	37	-7.80	8.7000	39	-4.30	8.8000	-3.50	[-7.44; 0.44]	9.3%
Liu 2020	100	-10.10	12.5000	97	-6.00	12.6000	-4.10	[-7.61; -0.59]	11.7%
Meurer 2019	14	-9.10	13.9000	16	-6.60	16.9000	-2.50	[-13.53; 8.53]	1.2%
Persell 2020	144	-8.30	13.8000	152	-6.80	13.7000	-1.50	[-4.63; 1.63]	14.6%
Skolarus 2018	41	-11.30	33.9000	32	-14.40	26.4000	3.10	[-10.73; 16.93]	0.7%
**Effect within subgroup (REM)**	**838**			**824**			**-2.74**	**[-4.03; -1.46]**	**86.8%**
Heterogeneity: $I^2 = 0\%, \tau^2 = 0, p = 0.9081$									
**Person Involvement**									
Kim 2016	52	-2.70	17.8000	43	-5.70	19.3000	3.00	[-4.53; 10.53]	2.5%
Li 2019	110	-5.50	15.0000	143	1.60	17.1000	-7.10	[-11.06; -3.14]	9.1%
Still 2020	30	0.55	18.2000	30	-2.17	19.7000	2.72	[-6.88; 12.32]	1.6%
**Effect within subgroup (REM)**	**192**			**216**			**-1.33**	**[-8.57; 5.91]**	**13.2%**
Heterogeneity: $I^2 = 73.5\%, \tau^2 = 28.2977, p = 0.0229$									
**Overall effect (REM)**	**1030**			**1040**			**-2.91**	**[-4.11; -1.71]**	**100.0%**
Heterogeneity: $I^2 = 3.7\%, \tau^2 = 0, p = 0.4082$									
Test for subgroup differences: $p = 0.7066$									

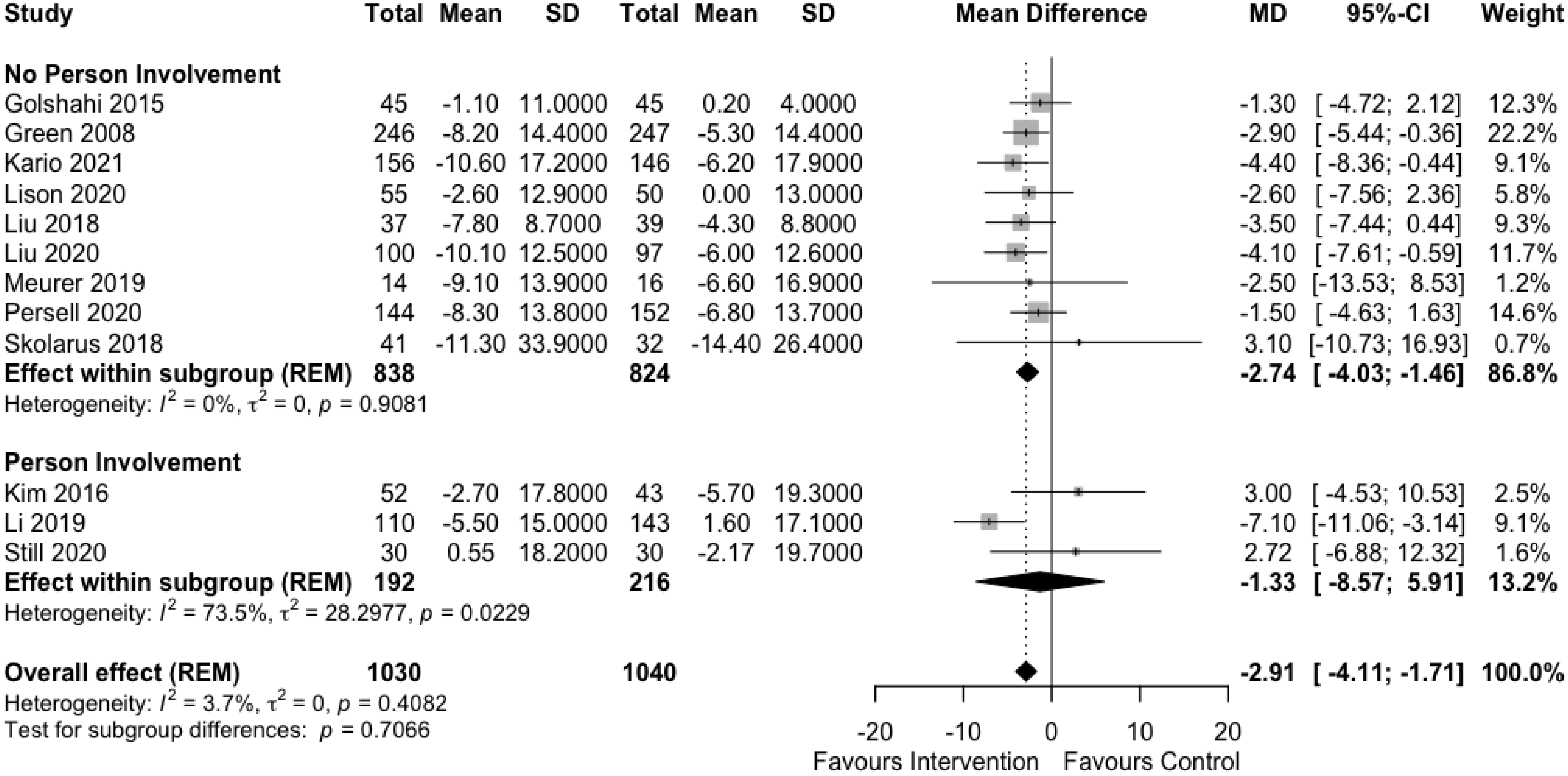

SDC 7. Sensitivity analysis of effect of bias risk on systolic blood pressure outcome

Study	Intervention Total	Intervention Mean	Intervention SD	Control Total	Control Mean	Control SD	MD	95%-CI	Weight
**Low Bias Risk**									
Green 2008	246	-8.20	14.4000	247	-5.30	14.4000	-2.90	[-5.44; -0.36]	22.2%
Kario 2021	156	-10.60	17.2000	146	-6.20	17.9000	-4.40	[-8.36; -0.44]	9.1%
Lison 2020	55	-2.60	12.9000	50	0.00	13.0000	-2.60	[-7.56; 2.36]	5.8%
Meurer 2019	14	-9.10	13.9000	16	-6.60	16.9000	-2.50	[-13.53; 8.53]	1.2%
**Effect within subgroup (REM)**	**471**			**459**			**-3.20**	**[-5.13; -1.26]**	**38.3%**
Heterogeneity: $I^2 = 0\%$, $\tau^2 = 0$, $p = 0.9239$									
**Unclear/High Bias Risk**									
Golshahi 2015	45	-1.10	11.0000	45	0.20	4.0000	-1.30	[-4.72; 2.12]	12.3%
Kim 2016	52	-2.70	17.8000	43	-5.70	19.3000	3.00	[-4.53; 10.53]	2.5%
Li 2019	110	-5.50	15.0000	143	1.60	17.1000	-7.10	[-11.06; -3.14]	9.1%
Liu 2018	37	-7.80	8.7000	39	-4.30	8.8000	-3.50	[-7.44; 0.44]	9.3%
Liu 2020	100	-10.10	12.5000	97	-6.00	12.6000	-4.10	[-7.61; -0.59]	11.7%
Persell 2020	144	-8.30	13.8000	152	-6.80	13.7000	-1.50	[-4.63; 1.63]	14.6%
Skolarus 2018	41	-11.30	33.9000	32	-14.40	26.4000	3.10	[-10.73; 16.93]	0.7%
Still 2020	30	0.55	18.2000	30	-2.17	19.7000	2.72	[-6.88; 12.32]	1.6%
**Effect within subgroup (REM)**	**559**			**581**			**-2.60**	**[-4.58; -0.61]**	**61.7%**
Heterogeneity: $I^2 = 35.3\%$, $\tau^2 = 2.4967$, $p = 0.1470$									
**Overall effect (REM)**	**1030**			**1040**			**-2.91**	**[-4.11; -1.71]**	**100.0%**
Heterogeneity: $I^2 = 3.7\%$, $\tau^2 = 0$, $p = 0.4082$									
Test for subgroup differences: $p = 0.6706$									

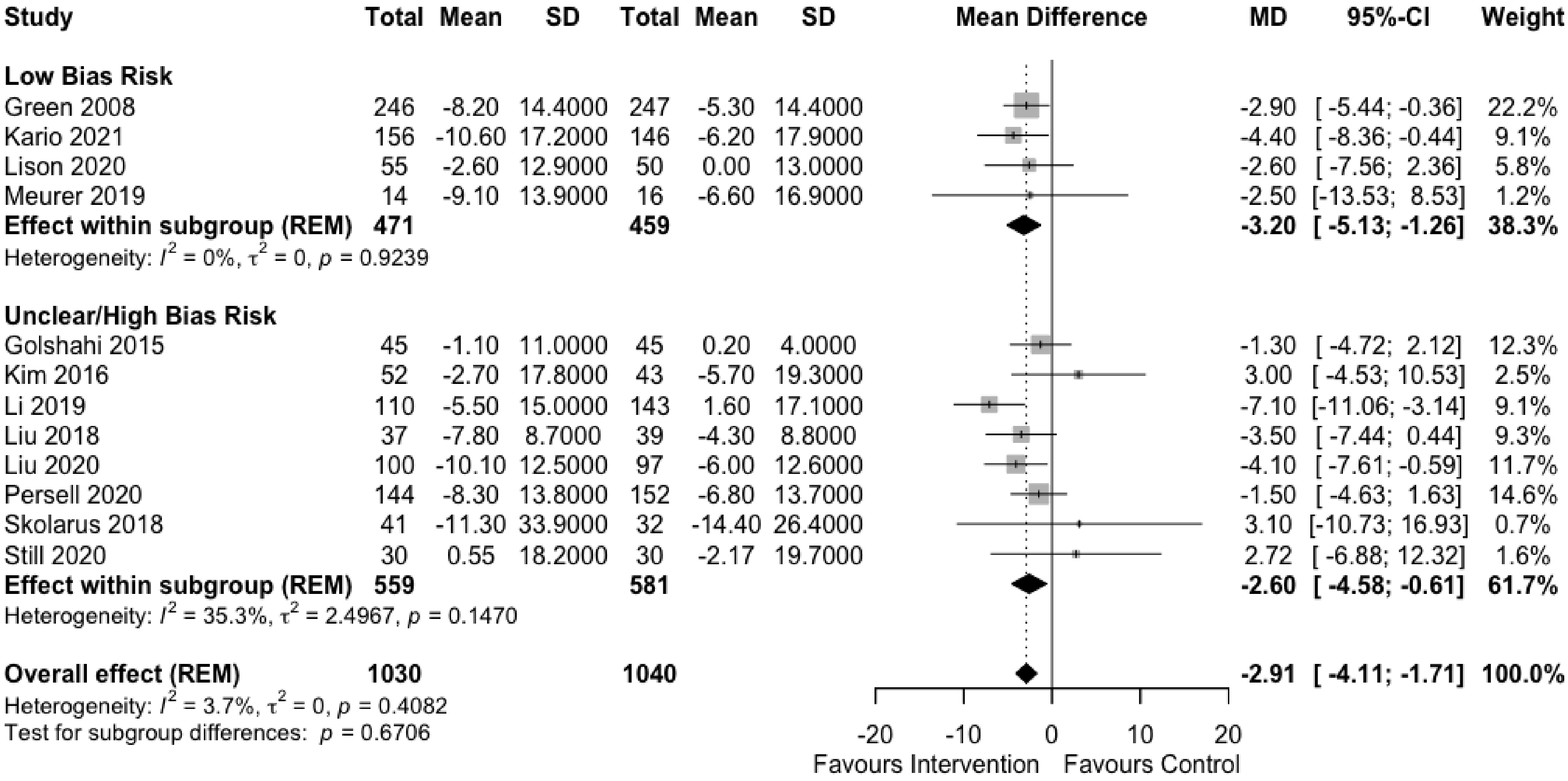

SDC 8. Funnel plot of studies included in the meta-analysis.

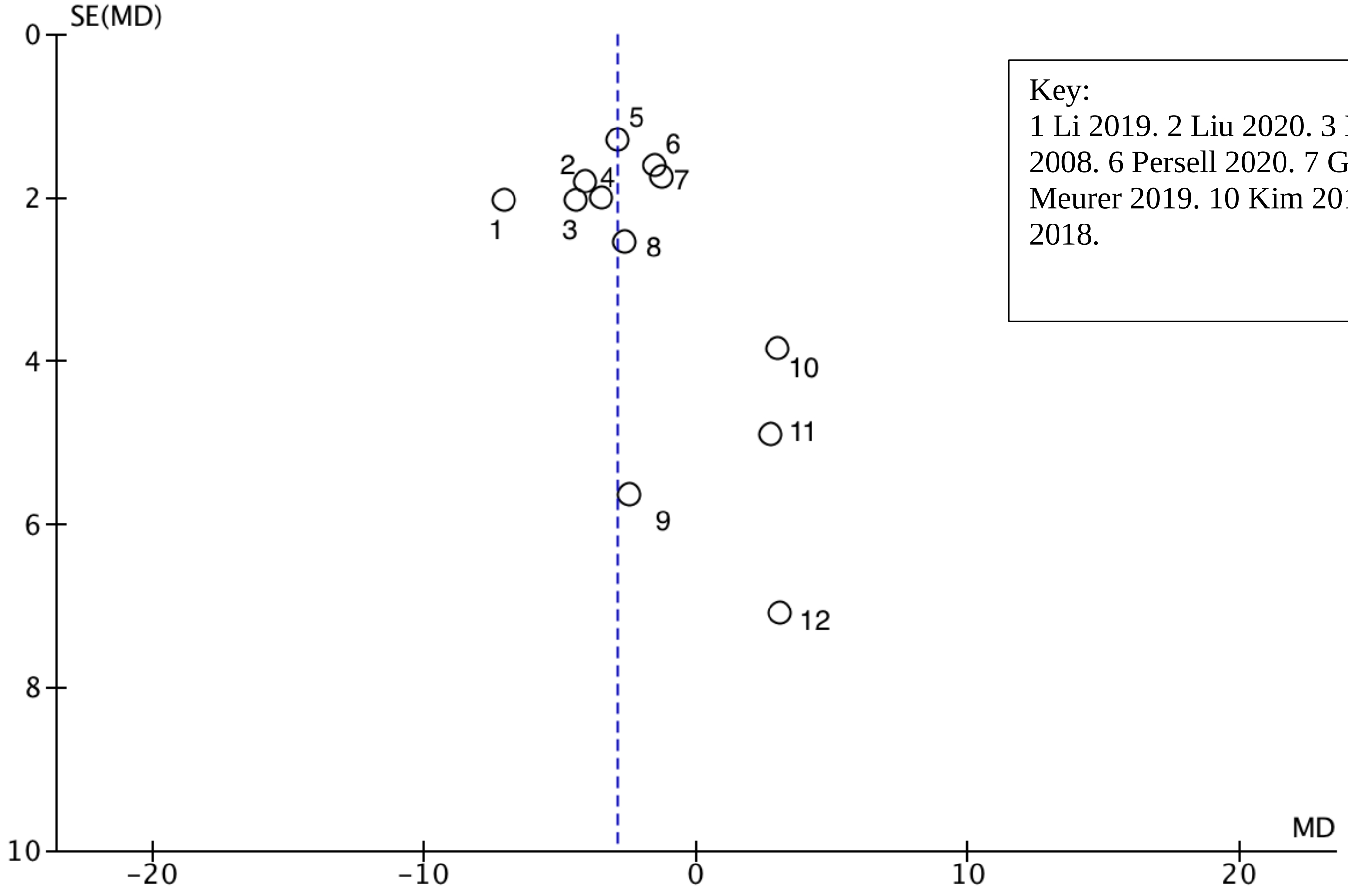

Key:
1 Li 2019. 2 Liu 2020. 3 Kario 2021. 4 Liu 2018. 5 Green 2008. 6 Persell 2020. 7 Golshahi 2015. 8 Lison 2020. 9 Meurer 2019. 10 Kim 2016. 11 Still 2020. 12 Skolarus 2018.

SE: Standard Error; MD: Mean Difference

SDC 9. Forest plot of the effect on diastolic blood pressure of digital interventions targeting lifestyle factors for hypertension.

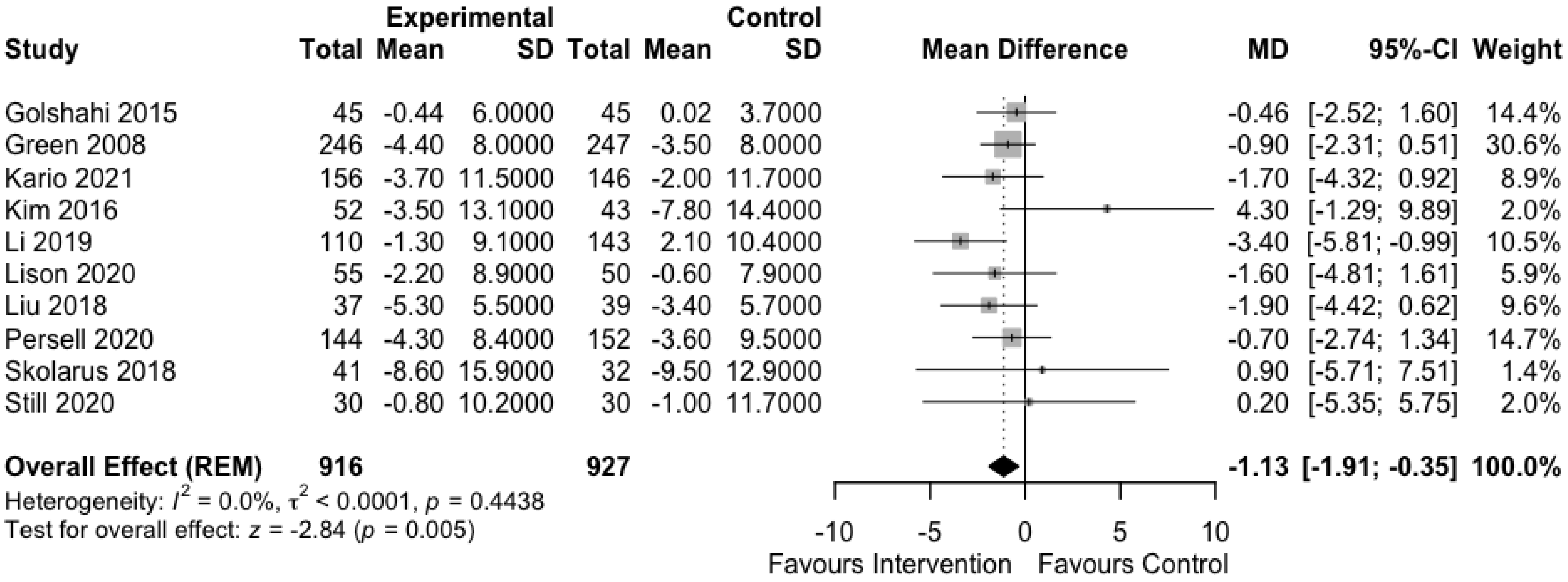

SDC 10. Summary of secondary outcomes reporting in included studies

Study	BMI (Kg/m^2)			Weight change			Physical activity			Salt intake			Diet			Smoking	
	**Unit**	**Between group comparison?**	**p-value**	**Unit**	**Between group comparison?**	**p-value**	**Unit**	**Between group comparison?**	**p-value**	**Unit**	**Between group comparison?**	**p-value**	**Unit**	**Between group comparison?**	**p-value**	**Unit**	**Between group comparison?**
Green, 2008[21]	Kg/m^2	No	-	VNR	-	-	% active based on questionnaire	No	-	VNR	-	-	VNR	-	-	VNR	-
Bennett, 2010[18]	Kg/m^2	No	-	Kg	No	-	VNR	-	-	VNR	-	-	VNR	-	-	VNR	-
Golshahi, 2015[33]	VNR	-	-	VNR	-	-	Minutes/day self-reported exercise	No	-	% with high salt intake on questionnaire	No	-	Vegetable intake times/day	No	-	% current smoker	No
Kim, 2016[24]	VNR	-	-	VNR	-	-	Exercise units/week based on questionnaire	No	-	VNR	-	-	VNR	-	-	Number of cigarettes per day	Yes
Liu, 2018[26]	VNR	-	-	VNR	-	-	Daily steps based on pedometer	Yes	0 74	VNR	-	-	Fruit servings/day	Yes	0 92	VNR	-
Skolarus, 2018[22]	VNR	-	-	VNR	-	-	VNR	-	-	VNR	-	-	VNR	-	-	VNR	-
Li, 2019[31]	VNR	-	-	VNR	-	-	VNR	-	-	VNR	-	-	VNR	-	-	VNR	-
Meurer, 2019[23]	VNR	-	-	VNR	-	-	VNR	-	-	VNR	-	-	VNR	-	-	VNR	-

Rehman, 2019 [30]	VNR	-	-	VNR	-	-	VNR	-	-	VNR	-	-	VNR	-	-	VNR	-
Borgstrom, 2020[27]	Kg/m^2	Yes	0 13	VNR	-	-	VNR	-	-	VNR	-	-	VNR	-	-	VNR	-
Jahan, 2020[29]	VNR	-	-	VNR	-	-	Unspecified	No	-	Food salinity checker	No	-	Unspecified	No	-	VNR	-
Lison, 2020[28]	Kg/m^2	No	-	VNR	-	-	Counts/minute based on accelerometer	No	-	VNR	-	-	VNR	-	-	VNR	-
Liu,2020[25]	VNR	-	-	VNR	-	-	Daily steps based on pedometer	Yes	0 02	24hour urinary sodium	Yes	0 57	VNR	-	-	VNR	-
Persell, 2020[20]	Kg/m^2	Yes	0 39	VNR	-	-	Minutes/week based on questionnaire	Yes	0 1	VNR	-	-	Consumption of processed meat, fried foods, sweetened beverages and candy d/week	Yes	0 22	VNR	-
Still,2020[19]	Kg/m^2	No	-	Lbs	No	-	VNR	-	-	VNR	-	-	VNR	-	-	VNR	-
Yun, 2020[32]	VNR	-	-	VNR	-	-	Metabolic equivalents of task activity based on questionnaire	Yes	0 23	VNR	-	-	VNR	-	-	VNR	-

Kario, 2021[34]	Kg/m^2	Yes	0 005	Kg	Yes	0 003	VNR	-	-	Salt intake based on salt check sheet	Yes	<0.001	VNR	-	-	VNR	-

VNR = value not reported

**p-value**
-
-
-
<0.001
-
-
-
-
